# An empirical approach to selecting community-based alcohol interventions: combining research evidence, rural community views and professional opinion

**DOI:** 10.1186/1471-2458-12-25

**Published:** 2012-01-10

**Authors:** Anthony Shakeshaft, Dennis Petrie, Christopher Doran, Courtney Breen, Robert Sanson-Fisher

**Affiliations:** 1 National Drug and Alcohol Research Centre (NDARC), University of New South Wales, Sydney, NSW 2052, Australia; 2 Economic Studies, University of Dundee, 3 Perth Road Dundee, Dundee DD1 4HN, UK; 3 School of Medicine and Health Science, University of Newcastle, Sydney, NSW, Australia

## Abstract

**Background:**

Given limited research evidence for community-based alcohol interventions, this study examines the intervention preferences of rural communities and alcohol professionals, and factors that influence their choices.

**Method:**

Community preferences were identified by a survey of randomly selected individuals across 20 regional Australian communities. The preferences of alcohol professionals were identified by a survey of randomly selected members of the Australasian Professional Society on Alcohol and Other Drugs. To identify preferred interventions and the extent of support for them, a budget allocation exercise was embedded in both surveys, asking respondents to allocate a given budget to different interventions. Tobit regression models were estimated to identify the characteristics that explain differences in intervention preferences.

**Results:**

Community respondents selected school programs most often (88.0%) and allocated it the largest proportion of funds, followed by promotion of safer drinking (71.3%), community programs (61.4%) and police enforcement of alcohol laws (60.4%). Professionals selected GP training most often (61.0%) and allocated it the largest proportion of funds, followed by school programs (36.6%), community programs (33.8%) and promotion of safer drinking (31.7%). Community views were susceptible to response bias. There were no significant predictors of professionals' preferences.

**Conclusions:**

In the absence of sufficient research evidence for effective community-based alcohol interventions, rural communities and professionals both strongly support school programs, promotion of safer drinking and community programs. Rural communities also supported police enforcement of alcohol laws and professionals supported GP training. The impact of a combination of these strategies needs to be rigorously evaluated.

## Background

Alcohol use is a leading cause of morbidity and mortality, accounting for 3.2% of deaths and 4% of all Disability Adjusted Life Years (DALYs) worldwide [1] and 0.8% of deaths and 2.2% of all DALYs in Australia [2]. Although this harm was estimated to have imposed an annual total social cost in Australia in 2004/05 of $15.3 million [3], a recent report estimates excessive drinkers impose costs of $13 billion on the community in out-of-pocket expenses, forgone wages and lost productivity, approximately $0.8 billion for hospital and child protection costs and $6 billion for intangible costs [4].

Historically, alcohol interventions have targeted individual-level risk factors associated with high rates of consumption and harm, such as age, gender, ethnicity and socio-economic status [5,6]. More recent interest has focused on identifying community characteristics that facilitate risky alcohol consumption and subsequent harm, for which community-level interventions are appropriate [7,8]. To date, however, the only community-level interventions that have at least some evidence for their effectiveness are media advocacy [9-14], enforced point-of-sale legislation [11,12,15,16] and increased police visibility [14,17].

The four Randomised Controlled Trials (RCTs) of community-based alcohol interventions, which represent the most methodologically rigorous evidence, have shown small decreases in only two outcomes: adolescent alcohol use [11,18,19]; and a reduction in availability of alcohol to youth [20]. Although there are time, resource and legislative constraints on the types of interventions that can be evaluated in a prospective community trial (eg. changing alcohol taxation rates is highly unlikely to be possible), there is clear capacity to test the effectiveness of a wider range of community-based interventions. Moreover, given these 12 studies [9-20] were conducted in only three countries (USA = 9 studies, Sweden = 2 studies and New Zealand = 1 study), with the most diverse culture being a native American study [18], there is a need to evaluate community-based alcohol interventions in a wider range of countries and cultures.

An evidence-based approach to selecting community alcohol interventions would combine research evidence with community and professionals' views [21]. Research evidence is least susceptible to bias, but results from well-controlled trials typically have limited generalisability [22]. Complementing research evidence with community and professionals' views is likely to improve the acceptability and implementation of interventions, particularly when they are involved in their design and implementation or when research evidence is limited [21]. Given the process of combining research evidence with community and professionals' views has been inadequate for community-based alcohol interventions [23], more effective alignment between these three components may improve their uptake and cost-effectiveness [24,25], which are critical factors given the apparent acceptability of community action to communities themselves [26].

Given the lack of evidence for community-based alcohol interventions and the high methodological rigour of RCTs, the largest cluster RCT of a community-based approach aimed at reducing alcohol-related harm ever undertaken internationally was conducted in Australia: the Alcohol Action in Rural Communities (AARC) project. This study comprised 20 rural communities (10 experimental and 10 controls) in New South Wales (NSW) and built on the previous largest trial of a community-based intervention, comprising six, non-randomised US communities [13]. AARC also represents the only prospectively planned economic evaluation of the alcohol-related community-action ever undertaken internationally, comprising a benefit-cost analysis. Although the primary outcomes will not be published until 2012, the AARC study provided an opportunity to identify the intervention preferences of rural communities and alcohol professionals, and the factors that influence their choices, and to compare those with existing research evidence to identify community-based interventions for empirical evaluation.

## Method

### Samples

#### Communities

The 20 rural communities that participated in the AARC project were selected because they had a population size between 5,000 and 20,000 (identified as the approximate optimal size for effective activation of community-based studies [27,28], were at least 100 km away from a major urban centre (population ≥ 100,000) and were not currently involved in another public health project to reduce alcohol harm.

#### Alcohol professionals

Professionals were selected from the approximately 350 members of the Australasian Professional Society on Alcohol and Other Drugs (APSAD), comprising counsellors, clinicians, policy professionals and researchers with a professional interest in the alcohol and other drugs field. In order to maintain confidentiality and independence from the researchers, the APSAD Secretariat agreed to mail the questionnaire in 2005, together with a pre-paid return envelope, to 200 randomly selected APSAD members who had listed alcohol as an area of interest. To optimise the response rate, APSAD re-sent the survey to the same 200 members after 2 weeks. De-identified responses were returned to the authors.

## Measures

### Rural community views

Rural community views were elicited from 2,977 surveys completed and returned in 2005 by randomly selected members of the 20 AARC communities (39% response rate) [29,30]. The sample comprised 18-62 year olds, selected using the age and gender distribution of these communities to optimise its representativeness [31]: 18 coincides with the minimum age for voting and legal drinking in Australia and those over 62 contribute relatively little to alcohol-related harm [32].

As part of the survey, respondents were asked to allocate a $1,000 budget (a reasonable household contribution over a lifespan and an easily divisible amount) across eight possible intervention types. This budget allocation exercise aimed to identify the types of interventions most commonly selected and the extent of support for them. The specific question was: *"Think about all problems related to alcohol in your community. These may include relationship difficulties, health issues, car accidents and crime. The next 3 questions ask you to consider what you would be prepared to do to reduce these problems. Your community is given $1,000 to spend on programs to reduce alcohol problems. It is your job to allocate this money. You can spend it all on one program (100%) or a combination of programs. Please enter answers in percentages and make sure it adds up to 100%." *Intervention options were: promotion of safer drinking through media and licensed venues (promote safer drinking); policies to reduce work-related drinking (workplace); information on alcohol harms provided by pharmacists (pharmacists); community-wide strategies to help local communities work together more effectively (community); advice from General Practitioners (GPs); school-based information (school); legal strategies, such as random breath testing and enforcing liquor licensing laws (police); and advice from hospital staff (hospital/emergency departments). These broad intervention areas were chosen because it was considered unlikely that the majority of the public would have knowledge about specific strategies. The order in which the first and last four interventions were presented was reversed in two different versions of the survey, to measure order response bias.

### Professionals' views

Similarly, professionals were asked to allocate a $100,000 budget (a reasonable government contribution and an easily divisible amount) to 23 interventions over 3 years (the maximum amount of time likely to be available to implement interventions in the AARC project) for a hypothetical rural community, the characteristics of which are summarised in Table 1. These characteristics were modelled on actual data from two rural communities in NSW, which were specified to standardise definitions given intervention preferences may change depending on community characteristics.

**Table 1:** Characteristics of a hypothetical rural community

Demographics		Medical and other services	
Population	12,000	No. of general practitioners (full-time)	14

Females: Males	1:1	No of GP practices	3

Proportion young persons (15-24 yrs)	13%	No. of drug and alcohol workers (full-time)	1

Proportion Indigenous Australians	5%	No. of hospitals (with 24 hr Emergency Department)	1

Distance to nearest large centre (more than 20,000 population)	170 km	No. of community pharmacies	2

Distance to nearest urban centre (more than 100,000 population)	400 km	Total no. of full time police & (no. of full time police on Highway Patrol)	14 (3)

Average annual wage/salary (Before tax)	$30,000	No. of high schools	3

Unemployment rate	8%	No. of licensed premises	10

**Crime and Health Statistics**	**Community average**	**State average**	

Assaults per 100,000 population	1100	1050	

Sexual assaults per 100,000 population	90	60	

Driving under the influence of alcohol or other drugs per 100,000 population	27	15.5	

Proportion of population who attended an emergency department in last 12 months	20%	13.5%	

Proportion of population who have had a heavy drinking day in the last 12 months	40%	35%	

Interventions were identified and categorised in a three step process. First, a list of potentially effective interventions was compiled from the existing literature relevant to community-based alcohol trials [6-20,33-40], excluding those not practically feasible for communities to implement (eg. alcohol taxation policy [41,42]. Second, this list of interventions was reviewed for its comprehensiveness and modified by five alcohol professionals. Third, similar interventions were grouped into categories to reduce the total number of interventions to a practical number (eg. codes of practice for hotels/bars and training programs for hotel staff were grouped together as 'promote safer drinking'). The interventions and intervention categories are listed in Table 2.

**Table 2:** List of selected interventions to which professionals could allocate a $100,000 budget

*Schools*
School-based programs



***Promote safer drinking***

Development of voluntary or mandatory codes of practice for hotels (eg. use of high impact plastic glasses, limiting the number of patrons present at any one time, making food and water available for free, free soft-drinks for designated drivers, banning promotions that encourage binge drinking, staggering closing times for different hotels, refusing entry after a set time, limiting take-away)

Expanded training programs for hotel staff (eg. responsible service of alcohol, how to avoid serving alcohol to intoxicated persons)

Media advocacy (regional television and radio, and local newspapers)



***Community programs***

Family-based interventions

Better integration between programs aimed at reducing alcohol harm and broader community programs, such as employment and education programs

Greater targeting of high-risk groups or environments (eg. Indigenous Australians, workplaces, youth and geographical areas)

Expansion of social work/community health roles to more effectively co-ordinate a range of services (eg. employment services, family support, financial advice, school counsellors) and improve their level of tailoring to the particular circumstances of individuals and families

Provision of self-help material and advice in the mail

Community drug and alcohol counsellors

Contributing resources to broader community development programs involving arts/culture and sporting/recreational events



***Police activity***

Promoting greater enforcement of existing liquor licensing laws by police (eg. underage drinking; not serving intoxicated patrons)

More effective random breath testing

More effective sentencing options for magistrates (eg. ignition locks and incarceration diversion programs)



***Training General Practitioners***

General practitioners



***Hospital/Emergency Departments***

Emergency Department (ED) staff

Hospital staff (other than EDs)

Supporting/establishing D&A clinics and residential rehabilitation

Ambulance officers



***Pharmacists***

Community pharmacists

Both the community and professionals' surveys also asked about respondents' personal and professional characteristics (specified in the data analyses section), to identify potential influences on their views.

### Data analyses

For both the communities and professionals, budget allocations erroneously reported as greater than 95% but less than 105% were proportionately re-scaled to ensure the total equalled 100%. Responses with errors outside this range were excluded.

The percentage of community and professional respondents who selected an intervention, and the average amount allocated to the intervention, are reported separately. Interventions selected by less than 25% of professionals were excluded from further analysis, as it was deemed unlikely that they would be included in an aggregated, optimal set of professionals' choices.

Tobit regression models were estimated to identify characteristics which explain differences in intervention preferences. Tobit models are appropriate because the budget allocation is constrained by a minimum of $0 (not selecting the intervention) and a maximum of either $1,000 (community survey) or $100,000 (professionals' survey). The outcome variable in all models is the average level of preference for an intervention (both the frequency with which it is selected and the budget amount allocated). The community preferences model was estimated with three levels of explanatory variables. First, the change in order of questions (order). Second, the extent of heterogeneity between communities (19 dummy variables). Third, individual-level factors: age; sex; education level; number in the household aged at least 14 years; frequency and quantity of alcohol consumption; having a family member/friend they perceive drinks too much; gross annual household income (from the mid-point of the selected income band); and dummy variables for gross annual household income greater than $78,000, don't know and prefer not to say. The professionals' preferences model was estimated with explanatory variables for whether the professional had lived or worked in a rural community, the number of years worked in the alcohol and other drugs field and whether the professional works for a government or other organisation.

The level of statistical significance was set at *p*≤0.01 for the rural community sample, due to its relatively large sample size, and *p ≤ *0.05 for the professionals' sample.

## Results

### Rural community views

#### Sample characteristics

The mean age of respondents was 42 yrs (*SD *12; range 18-71 yrs) and 45% of the sample was married. Mean years of education was 12, with 49% working full-time, 23% working part-time or casually, 12% on home duties, 10% retired, 4% students and 2% unemployed. A weekly household income of less than $500 was reported by 17%, while 19% had a weekly gross household income greater than $1,500. Indigenous and foreign born Australians accounted for 2% and 24% of the sample respectively. These sample characteristics were comparable to those from population census data, except for an over-representation of females and older people[31].

#### Intervention and resource allocations

**Figure 1:**
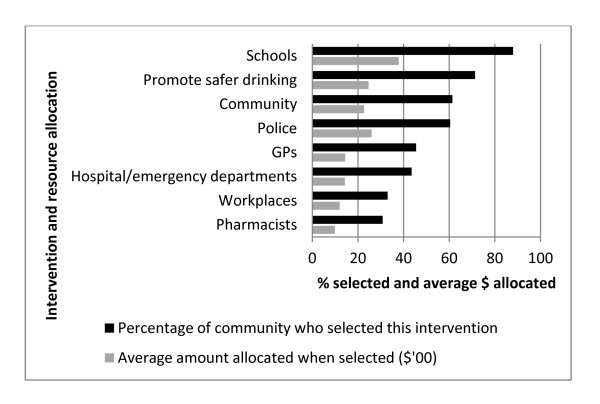
**Intervention and resource allocation preferences from a randomly selected sample of rural community residents (N = 2,811)**.

Of the 3,017 responses received, 148 did not answer the resource allocation question, 14 required re-scaling and 58 were excluded. The intervention and resource allocations of the 2,811 eligible respondents are summarised in Figure 1.

School programs were selected most often (88.0%) and allocated the largest proportion of funds when selected ($378), followed by promotion of safer drinking (71.3% selected, $245 allocated) and community programs (61.4% selected, $227 allocated). Police enforcement was the only other intervention selected by more than half the respondents (60.4% selected, $260 allocated).

#### Influences on rural community views

The Tobit regression showed that reversing the order in which the interventions were presented in the survey did not significantly change the frequency with which the top four interventions were selected (schools, promote safer drinking, community and police), although police enforcement was selected slightly more frequently than community-wide interventions. As summarised in Table 3, it also showed that intervention preferences are influenced by individuals' characteristics.

**Table 3:** Characteristics of rural community respondents that predict their alcohol-intervention preferences

Characteristics^a^	Intervention type Coefficient (robust standard error)							
	**School**	**Promote safer drinking**	**Community**	**Police**	**GP**	**Hospital/ED**	**Pharmacists**	**Workplace**

***Constant***	25.5* (5.21)	1.45 (4.65)	7.98 (5.20)	18.7* (5.83)	7.58 (3.61)	12.7* (3.75)	6.80 (3.35)	2.39 (3.86)

High frequency drinking	0.02 (0.07)	0.12 (0.06)	-0.12 (0.07)	-0.25* (0.07)	-0.01 (0.05)	0.16* (0.05)	-0.03 (0.04)	0.03 (0.05)

Family/friend drinks too much	-0.34 (0.76)	-0.16 (0.68)	2.28* (0.76)	-1.02 (0.86)	-0.13 (0.53)	-0.48 (0.56)	0.26 (0.56)	0.55 (0.57)

Female	2.43 (1.22)	-2.27 (1.08)	2.17 (1.22)	1.15 (0.86)	-0.17 (0.85)	2.32* (0.89)	0.12 (0.80)	-1.31 (0.91)

Age	0.14* (0.05)	-0.01 (0.05)	-0.10 (0.05)	-0.07 (0.06)	-0.09 (0.04)	-0.21* (0.04)	-0.19* (0.03)	-0.07 (0.04)

Education level	-0.52 (0.24)	0.86* (0.21)	0.14 (0.24)	-0.15 (0.27)	-0.29 (0.17)	-0.28 (0.17)	-0.30 (0.16)	-0.24 (0.18)

Gross annual household income > $78,000 pa	6.90* (2.28)	1.02 (2.03)	-2.08 (2.29)	-2.74 (2.56)	-3.48 (1.58)	-3.12(1.67)	-4.03* (1.50)	-4.12(1.71)

Prefer not to say income	3.45 (2.47)	0.30 (2.20)	-1.09 (2.47)	-0.76 (2.76)	-5.11* (1.72)	-1.77 (1.79)	-(1.72) (1.60)	-1.20 (1.82)

Community^b^	10.5	8.68	11.0	15.1*	6.33	11.2*	8.32	7.92

*Statistically significant where p ≤ 0.01

*^a^Neither high quantity drinking nor number of persons in the household aged *≥ *14 years were statistically significant predictors of intervention preference*

^b^Community is the maximum difference in average allocation preference between all twenty communities - significance is based on a joint test for significance of all community dummy variables

Hospital/Emergency Department (ED) interventions are most susceptible to response bias, being more strongly supported by frequent drinkers, females and some communities, and less strongly supported by older people. School-based programs were more strongly supported by older people and those with a gross annual household income greater than $78,000. Promoting safer drinking was supported by those with higher levels of education, while community-wide activities were supported by those who have a family member or friend whom they perceive drinks too much. GP-based interventions were supported by those who preferred not to state their income. Pharmacy interventions were supported less often by older people and those with a gross annual household income greater than $78,000, while police activity was supported less often by frequent drinkers. Workplace interventions were least susceptible to response bias.

## Professionals' views

### Response rate and sample characteristics

Of the 200 questionnaires mailed to APSAD members, five were returned with an incorrect address and 41 completed surveys were returned (21.0%). The average number of years respondents had worked in the alcohol and other drugs field was 14.6. The majority worked for a government organization (44%), 20% worked for a treatment organization, 17% worked for a university or research organization, 10% worked for a non-government organization, and 10% worked across multiple institutions. Sixty-one percent indicated that they had lived or worked in a rural town.

### Intervention and resource allocations

As summarised in Table 4, all 23 interventions were allocated at least some funds by at least one professional.

**Table 4:** Intervention and resource allocation preferences of professionals working in the alcohol and other drugs field in Australia (N = 41)

Interventions (in order of most often implemented)	Proportion of professionals who selected (%)	Average amount allocated when selected ($'000)
***Training General Practitioners***	***61.0***	***17.9***

***School programs***	***36.6***	***10.9***

***Community programs***	***33.8***	***15.3***

Family programs	26.8	9.1

Integration between programs	34.1	16.8

Targeting high risk groups	53.7	15.4

Expand social work services	43.9	21.1

Availability of self-help materials	4.9	5.0

Community drug and alcohol counsellors	31.7	14.0

Broad community development	41.5	25.9

***Promote safer drinking***	***31.7***	***8.7***

Harm reduction code of practice	46.3	9.3

Supply reduction code of practice	29.3	8.4

Training hotel staff	31.7	8.0

Regional radio	29.3	6.7

Regional television	26.8	11.9

Local newspapers	26.8	7.9

***Hospital/Emergency Departments***	***24.4***	***11.2***

Training Emergency Department staff	43.9	14.4

Training general hospital staff	24.4	7.7

Support drug and alcohol specialist clinics	17.1	16.5

Training ambulance officers	12.2	6.3

***Police activity***	***28.4***	***15.0***

Enforcement of liquor licensing laws	39.0	19.9

Random breath testing	34.1	17.4

More effective sentencing options	12.2	7.7

***Pharmacists***	***14.6***	***6.0***

Training GPs was selected most often (61.0%) and allocated the largest proportion of funds when selected ($17,900), followed by school programs (36.6% selected, $10,900 allocated), community programs (33.8% selected, $15,300 allocated), promoting safer drinking (31.7% selected, $8,700 allocated), hospital/emergency department interventions (24.4% selected, $11,200 allocated), police activity (28.4% selected, $15,000 allocated) and pharmacists (14.6% selected, $6,000 allocated).

### Influences on professionals' views

The Tobit regression showed no statistically significant relationships between professionals' characteristics and their intervention preferences, although there was a trend for professionals with more years of experience to allocate fewer resources to school-based interventions (*p ≤ *0.06).

## Discussion

The current evidence-base for community-level alcohol interventions comprises only very limited research evidence for the effectiveness of media advocacy [9-14], enforced point-of-sale legislation [11,12,15,16] and increased police visibility [14,17]. In the absence of sufficient research evidence, the principles of best-evidence practice advocate consideration of consensus view [21]. This study identified the intervention preferences of a random sample of rural community respondents, of which the four most commonly selected were: school-based interventions; promotion of safer drinking (codes of practice and training for the staff of licensed premises and media advocacy); community-wide activity (better integration between groups, more social work and counselling services and community development programs); and police activity (enforcement of liquor licensing laws and greater visibility). Professionals working in the alcohol and other drugs field rated two of those in their four most commonly selected interventions (community-wide activity and promotion of safer drinking) but also included training and support for GPs and hospital/ED staff.

### Combining research evidence with the views of rural communities and professionals

This study shows broad agreement between the views of rural communities and alcohol and other drug professionals about preferred strategies for reducing alcohol misuse and related harms: two of the four strategies selected by at least 50% of respondents to both surveys were the same, namely, promotion of safer drinking and community-wide activity.

Promotion of safer drinking generally refers to attempts to control drinking environments, through mechanisms such as codes of practice or training hotel staff and, more generally, promoting more responsible drinking in the community through increased awareness of alcohol harms through media advocacy. Although these views do broadly align with the limited research evidence for media advocacy [9-14], enforced point-of-sale legislation [11,12,15,16] and increased police visibility [14,17], they do not appear to adequately reflect the need for concomitant enforcement of codes of practice: increased police activity was rated fourth by communities and fifth by professionals. Given the concept of codes of practice in alcohol outlets are well ingrained internationally [43], it may be possible to improve the cost-effectiveness of this approach by increasing the sophistication of tailoring of police visibility and enforcement of point-of-sale legislation, beyond the typical targeting of high-risk times, such as weekend nights, community events and seasonal effects. Alternatively, this idea could be expanded to other defined settings where alcohol may be regularly consumed, such as workplaces. Although many workplaces do have alcohol policies and counselling programs in place, these could be strengthened to reflect the high level of workplace-related alcohol harm [44]. There also appears to be potential to further utilise media advocacy: data on alcohol consumption and harms that is specific to a community could be provided to communities via the media and key stakeholders, as a behaviour change strategy that has been shown to be effective in promoting behaviour change among individuals [45] and health care professionals [46].

The apparent popularity of community activity reflects support for broad-based services, such as an increased number of counselling type services (family interventions, social work services, drug and alcohol counsellors) and community development programs (sports and arts facilities). These may reflect increased awareness of the socio-economic determinants of health [47] and the negative impact of alcohol misuse on people other than drinkers themselves [4], although improvements in community development programs need to be independent of alcohol industry advertising and support, to avoid deleterious impacts from increased alcohol advertising and supply. Community activity also encompassed the idea of improved integration between various initiatives to potentially improve their combined effect across the community. This is analogous to the introduction of random-breath testing in Australia, the effectiveness of which is attributable to the combined effect of the testing itself, along with a sustained awareness-raising media campaign, high visibility of testing and enforced consequences for being caught [48]. Despite the popularity of this idea with rural communities and professionals, and its centrality to the concept of community-wide interventions [7,26], there is little research evidence to date for its likely cost-benefit.

These rural communities ranked school-based interventions as their most preferred strategy, although the research evidence for its effectiveness in changing young people's drinking behaviour is equivocal [38,49-52]. Given its popularity with these rural communities, however, it is highly likely that the acceptability to a community of any type of community-wide approach would depend on the inclusion of a school-specific intervention. Given the lack of evidence, it seems reasonable that such an intervention could have a harm-reduction focus (that is, equipping young people with strategies for staying safe when they are drinking), in addition to attempting to reduce the frequency and quantity of their drinking.

The majority of alcohol and other drug professionals also selected greater support and training for GPs and hospital/ED staff. Although the popularity of GP interventions most likely reflects the wealth of evidence for the cost-effectiveness of screening and brief intervention delivered in primary care [53], it is unclear if increased GP effort would be effective across a whole population, that is, if GP-based interventions are a cost-beneficial community-level strategy. Increasing hospital/ED-based effort is problematic because of the time and resources restrictions, especially in ED settings [34]. Nevertheless, given evidence that the proportion of risky drinkers among ED patients is significantly higher than in the general community [54] and that those who report higher rates of consumption also report worse health [30], interventions delivered in health settings seem appropriate and there is some evidence for their effectiveness [54]. Screening and brief intervention (SBI) for alcohol could also be implemented in a wider variety of settings, particularly as SBI has evidence for its cost-effectiveness [55] and does not require a high-level of technical skill to implement. Community pharmacies have been recognised as one relatively novel and promising health setting in which SBI could be delivered [56], although there have been only two intervention outcome studies, the results of which were equivocal [57,58]. Both communities (Figure 1) and professionals (Table 4) ranked pharmacy-based interventions last. Some of the practical limitations of time and resources constraints could potentially be addressed by simultaneously offering internet-based SBI, evidence for which is emerging [59].

### Methodological considerations

The methodological limitations of this study are unlikely to have substantially impacted on the results. Although the response rate of 39% may have restricted the extent to which the responses are representative of all community members, it is higher than for the 2004 (34.1%) and 2007 (33.2%) Australian national household drug and alcohol surveys [60,61]. While females and older people were over-represented, the Tobit regression results show their preferences effectively counteracted each other for the intervention type most susceptible to bias: a hospital/ED intervention was supported more by females and less by older people. School and pharmacy interventions were supported more and less frequently by older people, but given these were easily the most and least supported strategies overall, the responses of older people are unlikely to alter the ordinal preferences of these extreme interventions. The other Tobit regression results were as expected, providing reasonable evidence for the face-validity of both consumer and professionals' preferences. Frequent drinkers prefer health, rather than law enforcement, interventions, higher income earners are more likely to prefer school-and pharmacy-based interventions, those who have a relative or friend who they think drinks too much are more likely to select community programs, more educated respondents preferred media advocacy strategies and preferences for hospital/ED interventions varied by community.

The lack of any statistically significant predictors of professionals' views may reflect the abstract nature of the budget allocation task or the relatively large number of potential interventions. However, the community characteristics were both specified and based on actual rural communities and the majority of professionals agreed or strongly agreed that the budget allocation was a realistic task facing those trying to reduce alcohol related harm (66.0%), and expressed confidence that their allocation would reduce alcohol related harm (58.5%). It is most likely this finding reflects the reasonable expectation that professionals' views are less susceptible to bias than those of consumers. Although the 21% response rate to the professionals' survey creates some uncertainty about the generalisability of their views, and we were unable to obtain any further information about non-responders due to APSAD's privacy concerns, the extent of any bias is most likely to be limited for a number of reasons: unlike community views, the Tobit regressions found no statistically significant predictors of professionals' views which would indicate substantial bias; their views are generally aligned with existing evidence (the majority supported GPs, about a third supported school, community or safer drinking interventions, and pharmacy interventions received the least support); they selected a range of strategies; and they worked for a variety of organisations. The less than ideal response rate to both surveys highlights the need for more effective methods of identifying professionals' and community views in the short-term and the need to increase the range of community-based alcohol interventions with demonstrable evidence for their effectiveness over the longer term.

## Conclusion

Although the research evidence base for effective community-level interventions is currently insufficient, the views of rural communities and professionals suggest a number of interventions that could be implemented and evaluated in combination. Promotion of safer drinking could explore more sophisticated tailoring and feedback of alcohol harm data, be expanded to other defined settings where drinking occurs regularly and increase the use of media advocacy. Community wide activity could improve integration between initiatives to avoid duplication of effort, improve the sustainability of prevention activity and improve its cost-effectiveness. School-based interventions appear important to rural communities, but may be better focusing on providing young people with harm reduction strategies, given the lack of evidence for school-based programs substantially modifying young people's drinking behaviours, while SBI could be offered in a range of health care settings and via the internet, especially for rural and remote communities where issues of privacy and limited resources may be more acute. In improving the existing evidence-base, it is critical that whichever combination of interventions is selected, their impact is quantified using methodologically rigorous evaluation designs and measures [8,62].

## Competing interests

The authors declare that they have no competing interests.

## Authors' contributions

AS, CD and RSF conceived the study, reviewed the draft surveys and refined the data analyses plans. DP and CB took primary responsibility for developing, designing and implementing the surveys and statistical methods. AS, DP and CB drafted the manuscript. All authors actively commented on the manuscript and approved the final version.

## Pre-publication history

The pre-publication history for this paper can be accessed here:

http://www.biomedcentral.com/1471-2458/12/25/prepub
